# Linking Neurocardiovascular Responses in the Active Stand Test to Adverse Outcomes: Insights from the Irish Longitudinal Study on Ageing (TILDA)

**DOI:** 10.3390/s25113548

**Published:** 2025-06-04

**Authors:** Feng Xue, Roman Romero-Ortuno

**Affiliations:** 1 Discipline of Medical Gerontology, School of Medicine, Trinity College Dublin, D02 PN40 Dublin, Ireland; romeroor@tcd.ie; 2 The Irish Longitudinal Study on Ageing (TILDA), Trinity College Dublin, D02 PN40 Dublin, Ireland

**Keywords:** neurocardiovascular response, active stand, adverse health outcomes, continuous physiological monitoring, signal preprocessing, neurovascular, cardiovascular, statistical parametric mapping, brain oxygenation, tissue saturation index (TSI), blood pressure

## Abstract

Background: This study aimed to investigate the neurocardiovascular responses during an Active Stand (AS) test, utilizing both pre-processed and raw signals, to predict adverse health outcomes including orthostatic intolerance (OI) during the AS, and future falls and mortality. Methods: A total of 2794 participants from The Irish Longitudinal Study on Ageing (TILDA) were included. Continuous cardiovascular (heart rate (HR), systolic (sBP), and diastolic (dBP) blood pressure) and near infra-red spectroscopy-based neurovascular (tissue saturation index (TSI), oxygenated hemoglobin (O_2_Hb), and deoxygenated hemoglobin (HHb)) signals were analyzed using Statistical Parametric Mapping (SPM) to identify significant group differences across health outcomes. Results: The results demonstrated that raw (unprocessed) signals, particularly O_2_Hb and sBP/dBP, were more effective in capturing significant physiological differences associated with mortality and OI compared to pre-processed signals. Specifically, for OI, raw sBP and dBP captured significant changes across the entire test, whereas pre-processed signals showed intermittent significance. TSI captured OI only in its pre-processed form, at approximately 10 s post-stand. For mortality, raw O_2_Hb was effective throughout the AS test. No significant differences were observed in either pre-processed or raw signals related to falls, suggesting that fall risk may require a multifactorial assessment beyond neurocardiovascular responses. Conclusions: These findings highlight the potential utility of raw signal analysis in improving risk stratification for OI and mortality, with further studies needed to validate these findings and refine predictive models for clinical applications. This study underscores the importance of retaining raw data for certain physiological assessments and provides a foundation for future work in developing machine-learning models for early health outcome detection.

## 1. Introduction

Orthostasis, defined as the body’s physiological response to transitioning to an upright posture, is essential for maintaining blood pressure stability and ensuring adequate blood flow to vital organs [1,2]. However, in some individuals, this regulatory mechanism can become compromised, resulting in a condition known as orthostatic intolerance (OI) [3]. OI is characterized by a spectrum of symptoms, including light-headedness, dizziness, visual disturbances, syncope or near-syncope, palpitations, and fatigue when assuming an upright position [4,5]. These symptoms are often a consequence of insufficient compensatory responses needed to sustain cerebral perfusion during upright posture [6,7]. Furthermore, conditions such as vestibular disorders [8] and functional disorders [9] may also contribute to the development of OI. The variability in symptoms associated with OI presents significant health challenges, affecting not only the physiological well-being of affected individuals but also their daily functioning and overall quality of life [10].

The active stand (AS) test has emerged as a valuable clinical tool for assessing OI by evaluating neurocardiovascular responses during the controlled transition from lying to standing [11]. Recently, near-infrared spectroscopy (NIRS) has been integrated into the AS test to monitor frontal lobe oxygenation, providing deeper insights into cerebral perfusion dynamics during orthostatic stress [12,13]. NIRS allows for the continuous, non-invasive measurement of oxygenated hemoglobin (O_2_Hb) and deoxygenated hemoglobin (HHb) concentrations within cerebral tissue [14,15]. The measurement of frontal lobe oxygenation via NIRS during an AS test can serve as an important indicator of cerebral perfusion adequacy in response to the orthostatic challenge [16,17].

One commonly utilized NIRS parameter for monitoring cerebral tissue oxygenation in clinical research is the Tissue Saturation Index (TSI), which is derived from the ratio of O_2_Hb to total hemoglobin concentration (O_2_Hb + HHb) [18,19,20]. TSI reflects the proportion of hemoglobin that is oxygenated, representing the balance between oxygen supply and consumption in the cerebral tissue [21]. Both TSI [22,23,24] and O_2_Hb [25,26,27] are commonly employed for monitoring cerebral tissue oxygenation; however, TSI is often preferred as it accounts for fluctuations in total hemoglobin concentration, arguably providing a more comprehensive evaluation of cerebral oxygenation [28,29]. Nevertheless, recent findings by Mol et al. [30] have raised concerns regarding the reliability of TSI for assessing cerebral oxygenation. These concerns stem from potential issues with the validity of assumptions required for calculating TSI, such as the assumption of homogeneity in brain tissue, which may not always hold true [31,32].

In analyzing data from the AS test, researchers commonly use both pre-processed and unprocessed signals to capture distinct aspects of neurocardiovascular responses. Pre-processed data, where baseline values are subtracted from the original signals, highlight relative changes from baseline and allow for a clearer view of how physiological responses deviate from a resting state. This approach can enhance the interpretability of subtle shifts in parameters such as blood pressure and brain oxygenation. However, unprocessed signals retain raw, absolute values and may reveal important features that pre-processing might obscure. Analyzing unprocessed data could thus provide a more comprehensive picture, capturing essential details in signal variability that may be crucial for identifying individuals at risk of adverse outcomes.

Statistical parametric mapping (SPM) is an analytical technique that applies Random Field Theory [33] to enable topological inferences across the entire trajectory of a dataset. Although its primary application has been in neuroimaging [34], SPM is versatile and can be applied to any signal that represents a continuous function of space or time. For one-dimensional trajectories, such as cardiovascular and neurovascular signals obtained during the AS test, SPM effectively quantifies group differences and identifies specific time points with statistically significant variations [35,36].

This study aimed to visualize and analyze neurocardiovascular responses during an AS test, focusing on the relationship between these signals and adverse health outcomes (OI during AS, and future falls and mortality). By using both pre-processed and unprocessed data from The Irish Longitudinal Study on Ageing (TILDA), we sought to identify group differences through Statistical Parametric Mapping (SPM) and to detect specific temporal regions that significantly differentiate these adverse outcomes. Additionally, we aimed to compare the effectiveness of TSI and direct measurements of O_2_Hb in characterizing cerebral oxygenation patterns associated with these outcomes.

## 2. Materials and Methods

### 2.1. Study Population

The cardiovascular and neurovascular data collected during the AS tests at Wave 3 of TILDA were utilized in this investigation. Wave 3 of TILDA took place from 2014 to 2015. During this wave, 6687 participants completed interviews in their homes, and 80% of them also underwent a health assessment, which was conducted either at their residence or at a dedicated health assessment centre at Trinity College Dublin. The AS test was performed exclusively at the latter location [37]. Participants under the age of 50 and those with missing AS values were excluded from the analysis. Longitudinal outcomes were collected from Waves 4 (2016), 5 (2018), and 6 (2022). Ethical approval was obtained for each wave from the Faculty of Health Sciences Research Ethics Committee at Trinity College Dublin, Ireland. All participants provided written informed consent, and the research was conducted in accordance with the principles outlined in the Declaration of Helsinki.

### 2.2. Active Stand

The AS is a standardized test used to evaluate cardiovascular and neurovascular responses triggered by the act of standing up. During the TILDA Wave 3 AS test [38], six continuous non-invasive physiological signals were monitored. Cardiovascular measurements, namely systolic blood pressure (sBP), diastolic blood pressure (dBP), and heart rate (HR), were captured using a digital artery photoplethysmography device. Simultaneously, NIRS-collected neurovascular data, namely O_2_Hb, HHb, and TSI of the left frontal lobe, were recorded. For each participant, the AS test involved a supine rest period of at least 5 min, followed by standing up as quickly as possible (with assistance from one person if needed) and remaining upright while being monitored for a period of 3 min.

### 2.3. Adverse Health Outcomes

This study used three health outcomes to evaluate the impact of orthostatic stress. The first outcome was OI, assessed immediately after participants stood up during the AS test by asking if they experienced symptoms. Participants were specifically asked to self-report if they experienced dizziness or light-headedness during the AS. Reporting any of these symptoms classified the participant as having OI. The second health outcome was the occurrence of any type of fall reported by participants from the time of the assessment up until Wave 6 of TILDA, which occurred seven years later. The third health outcome was mortality status by Wave 6, recording whether the participant had died by the time of the Wave 6 assessment [39].

### 2.4. Instrumentation

A Finometer device (Finometer MIDI, Finapres^®^ Medical Systems, Amsterdam, The Netherlands) was used to noninvasively measure reconstructed arterial pressure on a beat-to-beat basis. Utilizing the principles of photoplethysmography, the device captures the arterial pressure waveform at a sampling rate of 200 Hz through the volume-clamp method. This method involves maintaining the volume of the finger arteries constant throughout the assessment, which is achieved via optical sensors embedded in the finger cuff and a pneumatic control system [40]. The volume-clamp technique has been extensively validated and demonstrates strong agreement with both intra-arterial blood pressure monitoring [41] and the auscultatory method [42]. The Finometer also incorporates a position sensor that adjusts for hydrostatic pressure changes by compensating for the height of the finger relative to the heart.

NIRS is a non-invasive, non-ionizing technique used to monitor changes in O_2_Hb and HHb concentrations in different tissues [22,43,44]. Research has demonstrated strong correlations between NIRS measurements and other methods for assessing cerebral blood flow [45] and muscle activity [46]. The versatility and high temporal resolution of NIRS, available through various implementations such as time-resolved, frequency-domain, and continuous wave spectroscopy, highlight its broad utility for both research and clinical applications [47].

NIRS measurements are based on light absorption at different wavelengths, where approximately 850 nm corresponds to O_2_Hb and 760 nm corresponds to HHb [48]. Often, the combination of O_2_Hb and HHb is expressed as TSI, calculated as 100 times the ratio of O_2_Hb to total hemoglobin ((O_2_Hb/(O_2_Hb + HHb)) × 100) [49].

In this study, the PortaLite^®^ (Artinis Medical Systems, Elst, The Netherlands), a wireless NIRS device, was used to measure O_2_Hb, HHb, and TSI signals using the absolute concentration method based on spatially resolved spectroscopy. The device, equipped with an optical sensor containing one emitter and three detectors, provided real-time, multi-channel data transmission via Bluetooth^®^ at a maximum sampling rate of 50 Hz. Oxysoft v3.0.53 software was employed for setup, data recording, and exporting. The NIRS sensor was positioned approximately 2 cm above the left eye, at the FP1 location according to the 10–20 electrode system (3 cm lateral and 3.5 cm superior to the nasion) [50]. A blackout headband was used to cover the sensor and minimize the effect of ambient light on the recordings [38].

### 2.5. Signal Acquisition, Synchronization, and Preprocessing

A one-minute segment of the AS data, spanning from 20 s before standing to 40 s after, was selected for analysis in this study. The beat-to-beat cardiovascular signals from the Finapres^®^ MIDI were interpolated at a rate of 5 Hz, while the neurovascular signals recorded by NIRS were downsampled to match this frequency. To ensure proper synchronization, all signals were aligned using multiple manual markers placed throughout the recordings. The onset of standing (i.e., the moment participants began standing up from the supine position) was identified using an algorithm described by O’Connor et al. [51], which relies on the height sensor data from the Finapres^®^ MIDI. Baseline values for the cardiovascular and neurovascular signals were determined by averaging the readings obtained from 60 to 30 s before standing (during the supine resting position), in accordance with previous studies [11,38,52].

**Figure 1:**
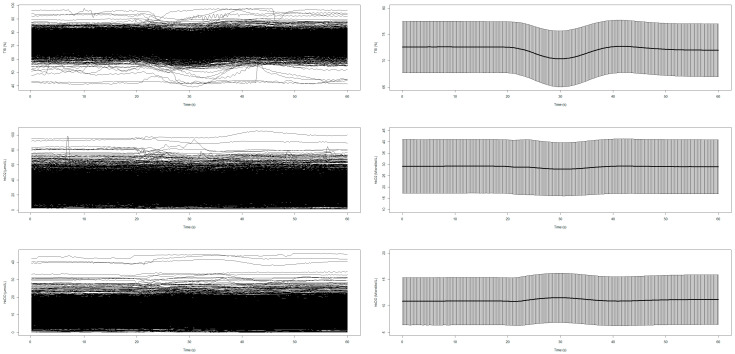
Unprocessed (raw) data of neurovascular signals, including TSI, O_2_Hb, and HHb, with corresponding mean ± SD plots shown on the right.

**Figure 2:**
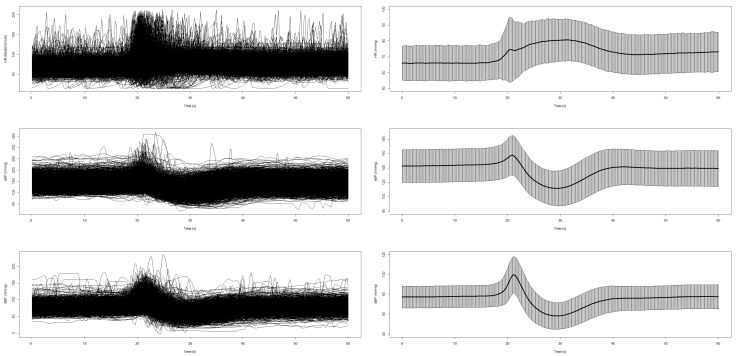
Unprocessed (raw) data of cardiovascular signals, including HR, sBP, and dBP, with corresponding mean ± SD plots shown on the right.

**Figure 3:**
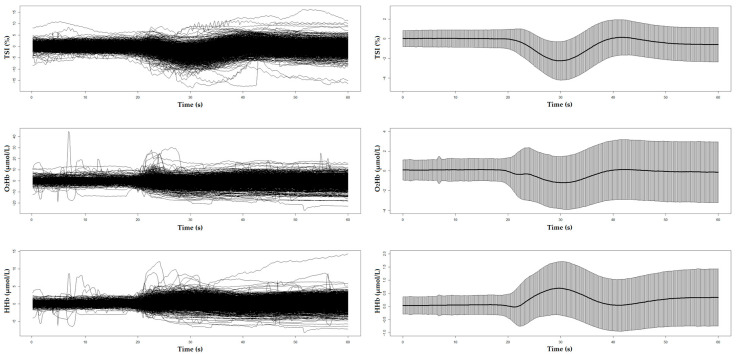
Pre-processed data of neurovascular signals, including TSI, O_2_Hb, and HHb, with corresponding mean ± SD plots shown on the right. Baseline values had been deducted from each signal for each participant, resulting in the relative changes shown in the plots.

**Figure 4:**
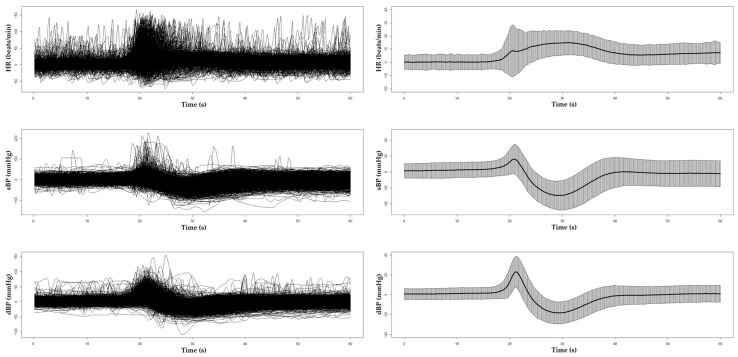
Pre-processed data of cardiovascular signals, including HR, sBP, and dBP, with corresponding mean ± SD plots shown on the right. Baseline values had been deducted from each signal for each participant, resulting in the relative changes shown in the plots.

Figure 1 and Figure 2 display the unprocessed (raw) signals in both neurovascular and cardiovascular signals, respectively. Raw signals reflect the direct, absolute values captured during the AS test, providing an unaltered view of each participant’s physiological response. These signals were also normalized (pre-processed) by subtracting individual baseline values and scaling them against the standard deviation of the baseline recordings. This normalization process, shown in Figure 3 and Figure 4 for neurovascular and cardiovascular signals, respectively, highlights relative changes from baseline.

### 2.6. Statistical Parametric Mapping

One-Dimensional Statistical Parametric Mapping (SPM1D) [53] is a Python/MATLAB package that has been employed to analyze various physiological traces [54,55,56]. Although several other software packages implementing the SPM methodology are readily available across various platforms (e.g., spmR, SPM12, and NIPY), SPM1D is currently the only package explicitly crafted for analyzing one-dimensional data, such as the time series recorded during the AS test [57].

### 2.7. Statistical Analyses

Descriptive statistics for the cohort and temporal analyses of the cardiovascular and neurovascular measures were carried out in R (version 4.0.5) using RStudio 2022.07.1+554 (Boston, MA, USA). For SPM analyses, the open-source package SPM1d 0.4 (http://www.spm1d.org/, accessed on 14 March 2025), which is dependent primarily on SPM8 (https://www.fil.ion.ucl.ac.uk/spm/, accessed on 14 March 2025), was used in MATLAB environment (R2020b, The MathWorks, Inc., Natick, MA, USA). Independent *t*-tests were conducted within SPM1d, which returns regions of significance in the form of *p* values. These values represent a continuous range over which the curve is identified as not consistent with random sampling. To reduce false positives and capture suprathreshold clusters [58] of likely clinical significance (i.e., continuous regions of at least 2 s of duration where at least one heartbeat would have been included), a statistical threshold of *p* < 0.001 was chosen [36].

## 3. Results

The study cohort consisted of 2793 participants, of whom 46.9% were men. The average age of the participants was 64.5 years. A total of 824 participants (29.5% of the cohort) experienced OI during the AS test. A total of 49.3% of the cohort (1378 participants) had at least a fall by Wave 6 of the TILDA data collection, which took place 6 years after the assessment. It was in the same time frame that 152 participants had sadly passed away, which counts for 5.4% of the entire cohort.

**Figure 5:**
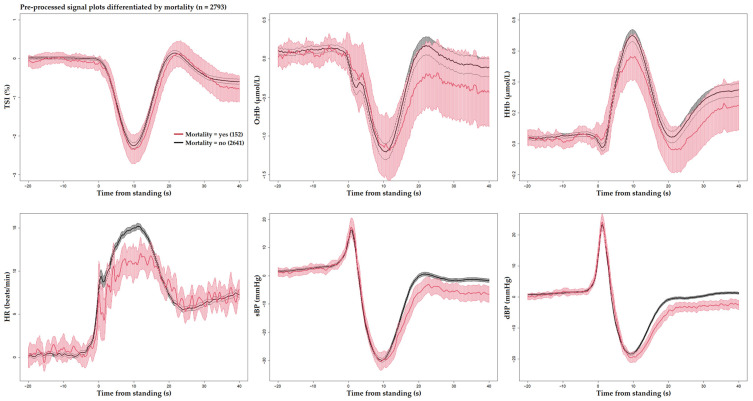
Visualization of neurocardiovascular responses across six pre-processed signals (baseline values subtracted from each original raw signal) during the AS test. Cerebrovascular signals (top row) are TSI, O_2_Hb, and HHb, while cardiovascular signals (bottom row) are HR, sBP, and dBP. For each signal, the mean response is represented by a solid line, with the 95% confidence interval shown as a shaded area. The **subsequently deceased group is shown in red** and the rest of the cohort in black. Standing occurs at 0 s in each plot.

**Figure 6:**
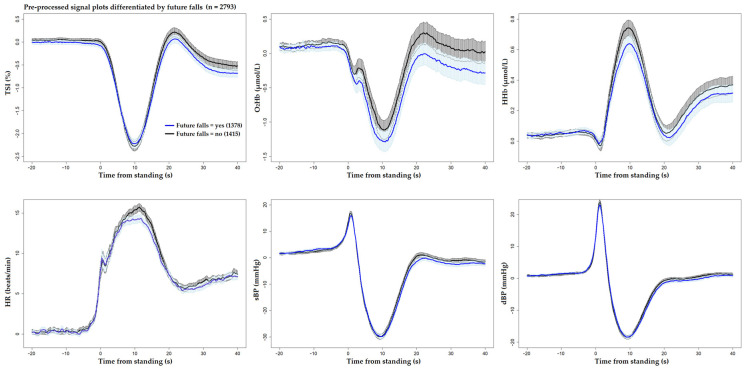
Visualization of neurocardiovascular responses across six pre-processed signals (baseline values subtracted from each original raw signal) during the AS test. Cerebrovascular signals (top row) are TSI, O_2_Hb, and HHb, while cardiovascular signals (bottom row) are HR, sBP, and dBP. For each signal, the mean response is represented by a solid line, with the 95% confidence interval shown as a shaded area. **The group who experienced future falls is shown in blue** and the rest of the cohort in black. Standing occurs at 0 s in each plot.

**Figure 7:**
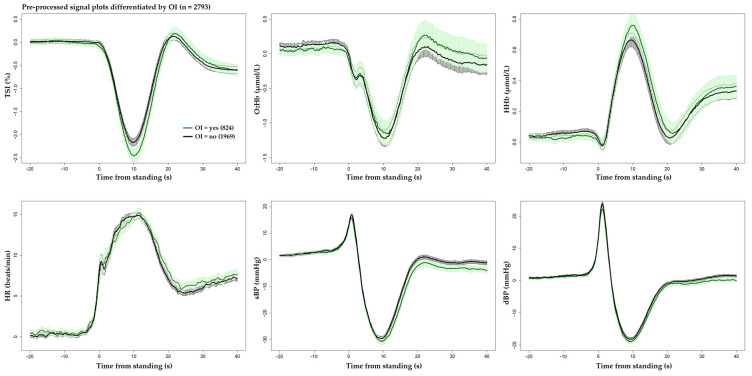
Visualization of neurocardiovascular responses across six pre-processed signals (baseline values subtracted from each original raw signal) during the AS test. Cerebrovascular signals (top row) are TSI, O_2_Hb, and HHb, while cardiovascular signals (bottom row) are HR, sBP, and dBP. For each signal, the mean response is represented by a solid line, with the 95% confidence interval shown as a shaded area. The group who **experienced OI during the AS is shown in green**, and the rest of the cohort in black. Standing occurs at 0 s in each plot.

**Figure 8:**
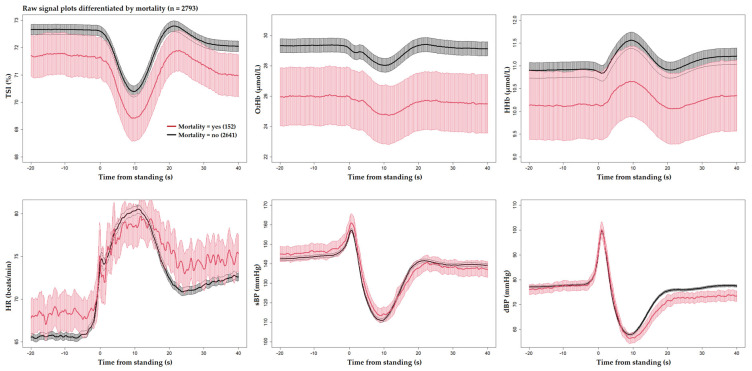
Visualization of neurocardiovascular responses across all six unprocessed signals (as recorded, without baseline adjustment) during the AS test. Cerebrovascular signals (top row) are TSI, O_2_Hb, and HHb, while cardiovascular signals (bottom row) are HR, sBP, and dBP. For each signal, the mean response is represented by a solid line, with the 95% confidence interval shown as a shaded area. The **subsequently deceased group is shown in red** and the rest of the cohort in black. Standing occurs at 0 s in each plot.

**Figure 9:**
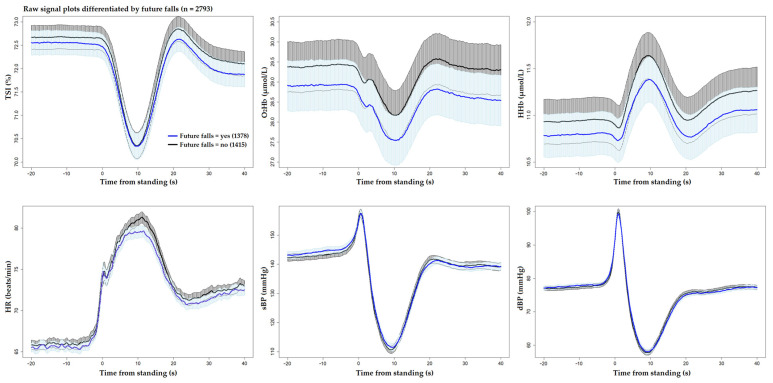
Visualization of neurocardiovascular responses across all six unprocessed signals (as recorded, without baseline adjustment) during the AS test. Cerebrovascular signals (top row) are TSI, O_2_Hb, and HHb, while cardiovascular signals (bottom row) are HR, sBP, and dBP. For each signal, the mean response is represented by a solid line, with the 95% confidence interval shown as a shaded area. **The group who experienced future falls is shown in blue** and the rest of the cohort in black. Standing occurs at 0 s in each plot.

**Figure 10:**
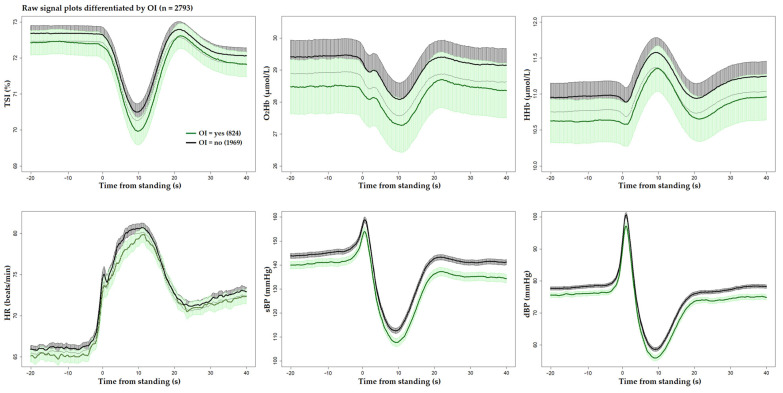
Visualization of neurocardiovascular responses across all six unprocessed signals (as recorded, without baseline adjustment) during the AS test. Cerebrovascular signals (top row) are TSI, O_2_Hb, and HHb, while cardiovascular signals (bottom row) are HR, sBP, and dBP. For each signal, the mean response is represented by a solid line, with the 95% confidence interval shown as a shaded area. The group who **experienced OI during the AS is shown in green**, and the rest of the cohort in black. Standing occurs at 0 s in each plot.

Figure 5, Figure 6 and Figure 7 display pre-processed signals for all six AS metrics, illustrating group differences in mortality, falls, and OI, respectively. Figure 8, Figure 9 and Figure 10 show the six raw (unprocessed) signals, highlighting group differences in mortality, falls, and OI, respectively.

**Figure 11:**
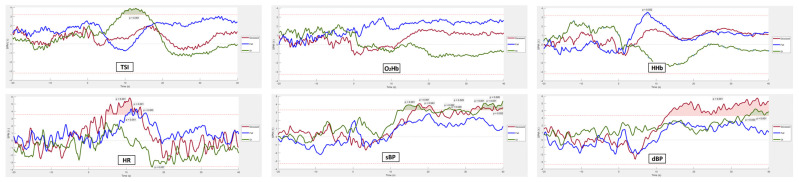
SPM analysis of all six pre-processed signals (baseline values subtracted from each original raw signal) during the AS test. Cerebrovascular signals (top row) are TSI, O_2_Hb, and HHb, while cardiovascular signals (bottom row) include HR, sBP, and dBP. For each signal, comparisons were made across the three outcome groups—deceased, falls, and OI—within the 60 s section of the AS test. Shaded regions above the dotted red lines indicate locations where statistical differences (*p* < 0.05) were observed between each outcome group and the rest of the cohort. Regions with statistically significant differences at *p* < 0.001 were considered potentially clinically significant (see text).

**Figure 12:**
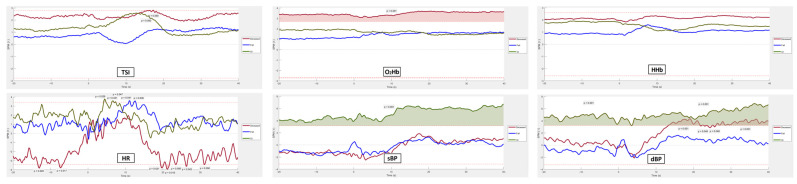
SPM analysis of all six unprocessed (raw) signals during the AS test. Cerebrovascular signals (top row) are TSI, O_2_Hb, and HHb, while cardiovascular signals (bottom row) include HR, sBP, and dBP. For each signal, comparisons were made across the three outcome groups—deceased, falls, and OI—within the 60 s section of the AS test. Shaded regions above the dotted red lines indicate locations where statistical differences (*p* < 0.05) were observed between each outcome group and the rest of the cohort. Regions with statistically significant differences at *p* < 0.001 were considered potentially clinically significant (see text).

Figure 11 and Figure 12 present the SPM analysis of neurocardiovascular responses during the AS test, comparing both pre-processed (Figure 11) and unprocessed (raw) signals (Figure 12) across the six metrics: TSI, O_2_Hb, HHb (cerebrovascular signals, top rows), HR, sBP, and dBP (cardiovascular signals, bottom rows). For each signal, statistical comparisons were conducted across three outcome groups—deceased, falls, and OI—within the 60 s post-stand period. Shaded regions above the dotted red lines indicate time points where statistical differences (*p* < 0.05) were observed between each outcome group and the rest of the cohort. In terms of regions with heightened significance (*p* < 0.001) which we flag as potentially clinically significant, we observed the following:

- OI. TSI captured OI only in the pre-processed data, around 10 s post-stand, while neither pre-processed nor raw O_2_Hb or HHb effectively captured OI. HR also showed no significant capture of OI in either form. sBP in the pre-processed data showed significant regions between 10 and 40 s after standing, whereas raw sBP effectively captured OI throughout the entire (pre-stand and post-stand) period. dBP showed brief significance in the last 5 s when pre-processed, but raw dBP was effective in capturing OI throughout the entire duration of the test.
- Future falls. No clear differences were observed between fallers and non-fallers across any of the six AS signals, whether pre-processed or raw.
- Mortality. For mortality, none of the pre-processed NIRS signals showed clear discrimination, while raw O_2_Hb effectively captured mortality throughout both the pre-stand and post-stand periods. The pre-processed HR distinguished mortality around 10 s post-stand, and pre-processed sBP showed brief significance around 20 and 30 s post-stand. Pre-processed dBP displayed clear significance in the continuous period after 10 s of standing. In contrast, raw HR and sBP did not differentiate mortality, although raw dBP continued to show significant discrimination in periods following 10 s of standing.

## 4. Discussion

In this study, we conducted SPM analysis on non-invasively collected, continuous cardiovascular and neurovascular signals recorded during an AS test in a large population-based sample (TILDA). Our primary objective was to compare the clinical relevance of pre-processed signals to their original, unprocessed counterparts by examining their associations with three adverse health outcomes. Additionally, we sought to evaluate the relevance of TSI, a commonly used derived variable, against directly measured O_2_Hb as an indicator of cerebral oxygenation under orthostatic stress. We used two longitudinal outcomes—mortality and falls—alongside OI, assessed immediately during the AS, as classification indices. These outcomes were selected due to their recognized clinical importance [59,60,61].

From our results, we observed that raw (unprocessed) signals generally demonstrated superior sensitivity in capturing significant physiological responses related to adverse outcomes, particularly OI and mortality, compared to pre-processed signals. For OI, raw sBP and dBP consistently captured OI throughout both the pre-stand and post-stand periods, while pre-processed signals only showed intermittent or brief significance. Notably, TSI was effective in capturing OI only in the pre-processed form, around 10 s post-stand, suggesting that specific signals may still benefit from preprocessing depending on the target outcome.

For mortality, raw O_2_Hb was effective in distinguishing between mortality outcomes throughout the entire AS period, while none of the pre-processed NIRS signals achieved this level of discrimination. This suggests that the absolute magnitude of the O_2_Hb signal may be able to capture participant features associated with future mortality, as opposed to using the pre-processed signals. For example, participants with lower absolute O_2_Hb levels, as seen in the mortality group in Figure 8, could have abnormal neurovascular coupling and impaired cerebral autoregulation [62]. This observation resonates with the theory that cerebral hypoxia, a condition where the absolute oxygen concentration in cerebral tissue falls below a critical threshold, is closely related to increased mortality [63,64,65]. Under hypoxic conditions, neurovascular coupling, a mechanism by which active neurons signal for increased blood flow to meet metabolic demands, can become dysfunctional, resulting in insufficient oxygen delivery to critical brain regions that supports neurovascular signal coupling [66]. Cerebral autoregulation, the brain’s ability to maintain consistent blood flow despite changes in systemic blood pressure, can be compromised during hypoxia, causing fluctuations in cerebral blood flow, exacerbating neuronal injury [67] and increasing the risk of mortality [68]. Furthermore, results suggest that a threshold value may exist in the O_2_Hb signal that differentiates individuals at risk of cerebral tissue hypoxia, although such a threshold has yet to be established in humans using NIRS technology. Additionally, pre-processed and raw dBP captured mortality-related differences throughout the post-stand period, while pre-processed HR and sBP demonstrated brief periods of significance post-stand.

In terms of fall risk, neither pre-processed nor raw signals showed clear distinctions between fallers and non-fallers, indicating that AS signals may not be useful for fall prediction without further refinement or additional measures.

These observations align with previous research underscoring the importance of blood pressure dynamics, particularly dBP, in predicting adverse health outcomes, including mortality. As a key indicator of vascular resistance and cardiovascular strain, dBP has been associated with heightened mortality risk in multiple epidemiological studies [69,70,71]. The ability of dBP to differentiate mortality risk, even in its unprocessed form, may indicate its fundamental role in autonomic regulation during orthostatic challenges, reflecting underlying pathophysiological changes that predispose individuals to poorer health outcomes [72,73,74]. This finding highlights the importance of retaining specific raw signal characteristics for risk stratification, particularly dBP, which may hold essential prognostic information in its unprocessed form. Further research is needed to explore whether these intrinsic differences in unprocessed dBP can be effectively utilized in clinical practice for early identification of individuals at high risk of mortality.

For OI, minor differences were observed in sBP and dBP during the recovery phase in the pre-processed data, while the unprocessed signals revealed a more pronounced deficit for the OI group, with statistical significance noted in both sBP and dBP over a broader period. This suggests that pre-processing may obscure critical signal features essential for differentiating individuals with OI, whereas unprocessed signals retain key information crucial for identifying significant hemodynamic changes associated with orthostatic stress. These findings align with the existing literature that underscores the role of subtle, dynamic blood pressure changes during orthostatic challenges as indicators of autonomic dysfunction and susceptibility to OI [75,76,77]. The reduction in signal variability caused by preprocessing may limit the ability to detect these differences, highlighting the importance of analyzing raw data in contexts where detailed cardiovascular responses are crucial.

In contrast, for the association with falls over a seven-year period, no statistically significant differences were observed in either the pre-processed or original unprocessed data across any cardiovascular parameters. This finding suggests that the cardiovascular responses measured during the AS test may not directly correlate with long-term fall risk. Instead, fall risk may be influenced by a complex interplay of neuromuscular, cognitive, and environmental factors, rather than autonomic cardiovascular responses alone [78,79,80]. Previous studies have also shown mixed results regarding the relationship between orthostatic blood pressure changes and fall risk, suggesting that effective fall prediction likely requires a multifactorial assessment approach that extends beyond cardiovascular metrics alone [81,82,83]. Therefore, the lack of significant findings in this area suggests that while cardiovascular parameters may be essential for understanding mortality and OI, they are likely insufficient as standalone predictors of long-term fall risk.

In the neurovascular domain, the differences associated with the three health outcomes were less pronounced. The only statistically significant finding was in the pre-processed TSI, approximately 10 s post-stand, in relation to OI. This suggests that neurovascular changes following the AS test may contribute to the development of OI, particularly in the early moments after the orthostatic challenge. Previous studies have shown that TSI reflects regional tissue oxygenation and is linked to autonomic regulation and cerebral perfusion during orthostatic stress [84,85]. The early response of TSI may provide insights into the compensatory mechanisms at play during the transition to standing, which, if impaired, can contribute to OI.

In contrast, markedly large differences were observed in raw O_2_Hb for individuals with a seven-year mortality outcome, spanning the entire 60 s of recording. This suggests that oxygen delivery and consumption, as reflected by O_2_Hb, may significantly differ between individuals at higher risk of mortality and those without, indicating potential impairments in neurovascular coupling or alterations in systemic oxygen dynamics. This finding aligns with studies on cerebral autoregulation, which have reported altered patterns of O_2_Hb in individuals with autonomic dysfunction during orthostatic challenges [86]. The sustained differences in O_2_Hb throughout the recording period indicate a prolonged disturbance in neurovascular function, which may be indicative of underlying pathophysiological conditions that contribute to increased mortality risk [87,88,89]. These findings collectively suggest that neurovascular signals, particularly TSI and O_2_Hb, may offer valuable insights into the physiological changes associated with mortality risk, highlighting the importance of detailed temporal analysis in understanding the dynamics of neurovascular regulation.

SPM was used to compare the time series data from different groups. To ensure the robustness of the findings and to reduce the chances of false positives, the threshold for statistical significance was set at *p* < 0.001, rather than the conventional *p* < 0.05. This more stringent threshold was intended to highlight only the most pronounced differences and to minimize the likelihood of detecting spurious associations that might arise due to the multiple comparisons inherent in time series analysis. By lowering the significance level, we aimed to increase the reliability of our findings, thereby ensuring that any observed differences were more likely to reflect true physiological distinctions rather than random variation. This approach is consistent with best practices in studies involving high-dimensional data, where controlling the false discovery rate is crucial to ensure meaningful interpretations [36,90,91,92]. Using a stricter significance threshold provides greater confidence in the observed results, particularly given the exploratory nature of the time-series comparisons across multiple health outcomes.

From a technical perspective, plotting all six continuous physiological signals in a synchronized manner, as shown in our figures, provided an efficient means of visually inspecting physiological responses across different groups during the AS test. This approach enabled us to propose potential connections between cardiovascular and neurovascular responses, facilitating the generation of hypotheses for future research. The inclusion of SPM analysis further enriched the stratified information within the assessment data by adding statistical significance, paving the way for a machine-learning-based knowledge pool that could support early detection of deteriorating health outcomes. This study represents a promising first step toward achieving this goal, with substantial potential benefits for healthcare professionals.

To our knowledge, the current study is the first to investigate the clinical relevance of TSI as an index for capturing the signature in brain oxygenation in the context of orthostatic challenges using SPM approach. A strength of the current study is the data-driven nature of the methodology that eliminates subjective influences on the health outcomes. Another strength is that the study is based on a large population-based sample, from which the physiological data were collected. This however precluded sub-analysis by sex, which could be of potential interest [93]. Another limitation is that two health outcomes, namely mortality and fall, used in this study are self-reported. We also acknowledge that while TILDA offers insights into the Irish community-dwelling context, it is important to replicate the research in various settings and countries to enhance the external validity of our findings.

## 5. Conclusions

Based on our findings, this study provides novel insights into the potential utility of continuous, non-invasive neurocardiovascular monitoring during an AS test to predict adverse health outcomes, including OI and mortality. The raw (unprocessed) signals, particularly O_2_Hb and blood pressure (BP), proved more effective than pre-processed signals in capturing significant physiological differences related to mortality and OI. Further studies are needed to validate these results across different populations and to refine predictive models that leverage these raw signal features for clinical applications.

## Data Availability

The datasets generated and/or analyzed during the current study are not publicly available due to data protection regulations but are accessible at TILDA on reasonable request. The procedures to gain access to TILDA data are specified at https://tilda.tcd.ie/data/accessing-data/, accessed on 1 March 2025.
